# The rise of eating disorders in Asia: a review

**DOI:** 10.1186/s40337-015-0070-2

**Published:** 2015-09-17

**Authors:** Kathleen M. Pike, Patricia E. Dunne

**Affiliations:** Departments of Psychiatry and Epidemiology, Columbia University, New York, USA; Department of Clinical & Counseling Psychology, Columbia University (Teachers College), New York, USA; New York State Psychiatric Institute, Unit 9, Rm. 5808, 1051 Riverside Drive, New York, NY 10032 USA

**Keywords:** Eating disorders, Asia, Culture, Prevalence, Body image, Dieting, Risk factor, Development, Globalization, Westernization, Nutrition transition, Urbanization, Anorexia nervosa

## Abstract

Once concentrated among adolescent Caucasian females in high-income Western countries, today, eating disorders (EDs) are truly global. Building upon previous work describing the rise of EDs among cultures in transition, we contextualize the emergence of EDs in Asia by locating this development within the broader discourse about the processes of change that have radically transformed Asian societies over the last three decades. By identifying where EDs are emerging in the region, and by examining their particular expression, our aim is to explicate a fuller story of the relationship between culture and eating disorders.

Much of the discussion of EDs in non-Western societies is predicated upon the assumption that an increase in EDs is the by-product of “Westernization”, the term used to describe the process by which increased cultural contact with the West results in the transmission of so-called ‘Western’ ideas and cultural norms to a non-Western culture. While the Westernization literature represents a historical anchor in our understanding of EDs in Asia, we propose that this analysis is incomplete in that societal change in the form of industrialization and urbanization occurring independently from, or in tandem with, “Western” influence are critical factors contributing to the rise of EDs in Asia. Further, our review of eating disorders in Asia suggests that an understanding of the diversity and distinctiveness of the individual countries and cultures that comprise ‘Asia’ is crucial to understanding the emergence and rise of EDs across this vast region, suggesting that eating disorders are not culture-bound or culture-specific, but rather culture-reactive. Taking into account both the historical influence of Western culture and the more contemporary effects of Asian industrialization and urbanization, key distinctions among respective Asian cultures expands our understanding of the development and expression of EDs globally.

## Introduction

In 1873, Sir William Gull in England and Charles Lasegue in France first described the “morbid mental state” of anorexia nervosa (AN) based on their clinical experience in the late nineteenth century, thus anchoring the modern study of eating disorders (EDs) in a specific cultural and historical context which has informed, but also limited, our understanding of these pathologies to this day [1, 2]. During the twentieth century, the study of EDs expanded to include a wider range of pathology, but it continued to be heavily concentrated in Western countries and in those countries with significant populations of European descent.

Cultural factors are essential to understanding the ways societies describe, diagnose and treat health conditions [3], and in the case of EDs, dramatic increases in prevalence began in the West during the mid-to-late 1960s in the wake of the counter-culture movement, and they continued to rise throughout the ensuing decades of the twentieth century [4]. Early conceptualizations of EDs characterized them as afflictions primarily affecting wealthy, white, educated, young women in industrialized Western nations [5]. Research focused on identifying the unique or particular features of society that increased risk for young women, and thus an enormous body of literature emerged documenting the pernicious effects of the ultra-thin beauty ideal that was marketed to young women not only as the epitome of female beauty, but furthermore, as an attainable ideal towards which all women should aspire. In the West, the spread of the thin ideal has been accompanied by the acceptance of the notion of ‘body instrumentality,’ which promotes the idea that improvement of the physical body via diet and exercise provides a pathway through which an individual may attain a certain level of control that is somehow essential to achieving his or her ideal self.

As the new millennium approached, EDs in non-Western cultures have steadily increased [4, 6–8], generating new data that shifted our frame of reference and necessitated an articulation of eating pathology that recognizes the heterogeneity of cultural expression and influence in a rapidly changing global context. Our conceptualization of EDs has evolved to encompass a more global perspective and more varied expression, as ED cases arising in the “East” have at times diverged from the “normative” reference provided by the West, in terms of risk and phenotype [9–12].

The emergence of EDs in Asia over the past three decades illuminates the complex interplay between culture and pathology. The proliferation of EDs across the Asian continent has coincided with an era of unprecedented growth and widespread social and economic transformation across much of the region [13–15]. Indeed, Asia now counts among its member nations some of the most advantaged and dynamic economic systems, which continue to evolve at a rapid pace [16–18]. By identifying where EDs are emerging within Asia, and by examining the particular expression of EDs in these contexts, our aim is to move beyond the notion that eating disorders are culture-bound and to explicate a fuller story of the relationship between culture and EDs.

Previous discussions of EDs in non-Western societies have focused on the phenomenon of Westernization. The study of westernization provides an important lens for understanding the rise of eating disorders in Asia; however, this analysis fails to recognize that amid globalization, countries in Asia are experiencing a rise in eating pathology as a result of multifaceted and profound cultural transformations [14, 19], driven by the processes of industrialization and urbanization occurring independently from, or in tandem with, “western” influence. These transformations include fundamental shifts in population demographics, food supply, global economies, gender roles, and the traditional family structure, The concurrent rise in EDs has given unique perspective to the overall field of study, revealing previously unknown manifestations of and documenting wide diversity in rates of prevalence within different national and social contexts, supporting the notion that there are multiple ways in which eating disorders are culturally-reactive but not necessarily culture-bound.

In the discussion below, we explore both the model of “westernization” and the ways in which Asia’s diverse and distinct cultural foundations are shaping and shifting the prevalence and expression of EDs as a result of the current impact of rapid economic growth, industrialization and urbanization, and intensifying globalization. This expanded understanding is also an opportunity – the drama unfolding in Asia challenges assumptions often made about EDs in non-Western countries and promises to enhance our understanding and treatment of EDs around the globe.

### Eating disorder prevalence, incidence & trends over time in Asia

It is crucial to recognize the size and scope of ‘Asia’, which constitutes the largest continent both in terms of landmass as well as population. Accordingly, the social, political, and economic conditions of countries in this region vary enormously, both historically and currently [20], and their distinct histories and trajectories of societal transformation inform our understanding of the role of culture in the incidence and expression of EDs.

Tellingly, the concept of an “Asian” people evolved not inside the region of Asia but in the US [21]. As immigrants from different countries in Asia arrived and settled in Hawaii and on the mainland, the dominant Caucasian American culture began indiscriminately lumping the successive waves of immigrants together, first under the label of the dominant immigrant nationality (i.e. ‘Chinese’ and then ‘Japs’) and later, referring to them as ‘Asians.’ The label ignores the diversity of the distinct cultures that characterize the region and is based largely on white, European-American stereotypical perceptions of homogeneity of individuals from the ‘East.’ This is reflected in studies of EDs in the West where the appellation of “Asian” is typically employed to describe individuals with non-Western heritage who hail from somewhere between India and Japan. Given the enormous diversity within the geographic region of Asia, such gross terminology falls short in capturing the nuance of culture in the etiology of EDs in the region.

Thus, it comes as no surprise that the study of EDs in Asia did not actually begin in “Asia” per say, but rather, began with studies of ‘Asians’ – immigrants from Asia and their descendants who were residing in industrialized, Western countries. Among these were studies of Asian schoolgirls in Great Britain [22–28] and of refugees from Southeast Asia in the U.S. (e.g. Kope & Sack, 1987) [29]. In Asia proper, a handful of cases of EDs were reported in select countries prior to the 1990s, including Malaysia [30], Singapore [31, 32], India [33], and among Chinese in Hong Kong (then under British sovereignty) [34–36], but on the whole, EDs were largely absent from Asia [4], with the exception of Japan, a country in which EDs arrived on the scene in the mid-1970s [9, 37]. By the close of the 20th century, disordered eating attitudes and behaviors increased dramatically across Asia’s high-income populations of young females, with clinical EDs proliferating beyond Japan to Singapore, Hong Kong, Korea, and Taiwan [4, 20, 35, 38–41].

Significantly, the order by which EDs have spread among Asian countries closely tracks the so-called “Asian miracle” of economic transformation as it extends across the region [16, 19]. Which is to say, Japan has led the pack, followed by the economies of Hong Kong, Singapore, Taiwan, and South Korea; then a second wave comprised of the remaining Association of Southeast Asian Nations (ASEAN) countries – Philippines, Malaysia, Indonesia, and Thailand; and lastly, China and Vietnam. Mirroring this pattern, EDs were initially found less often in less developed, poorer Asian countries such as China, India, Thailand, Indonesia, the Philippines [20], Cambodia, Myanmar, Laos, and Vietnam [39]. Expanding our scope to include Pakistan and Fiji adds evidence pointing toward a marked increase in the presence of EDs throughout the region varying directly with economic growth and concomitant industrialization and urbanization.

In sum, today, many Asian countries report EDs [4, 42–46] – as these countries have grown more industrialized and globalized, EDs have followed, and the gap is closing between several Asian countries and the West with regards to both clinical pathology, as well as more widespread disordered eating, weight and shape concerns, and dieting behaviors [39, 47]. Even in Asian countries where EDs are believed to still be less prevalent than in the West, comparative studies have emerged documenting eating attitudes and body dissatisfaction levels that are similar to or worse than those reported by individuals from Western countries [39, 48–51].

### EDs in Asia explained through the lens of “Westernization”

While the ubiquity of McDonald’s franchises across the Asian continent is a conspicuous sign of Western influence on Asia, its impact extends well beyond rench fries. In particular, the West has played a substantial part in Asia’s economic development, through various channels including foreign investment, a rapidly growing business community, technological advances which entwined global economies and financial markets, and the arrival of numerous US and European companies on Asia’s shores, at first largely as a result of their outsourcing manufacturing operations to the region, but eventually, in pursuit of profiting from an increasingly powerful ‘Asian’ consumer market. As a consequence of the West’s growing influence in the region, Asian urban populations were increasingly exposed to Western culture, and specifically, to Western media, which frequently forms the cornerstone of explanations proffered for the emergence and spread of Eds in Asia (such as Westernization). According to this perspective, increasing exposure to ‘the West’ transmits a ‘thin body ideal’ and imparts Western notions of body instrumentality to non-Western societies, which in turn fosters growing body dissatisfaction, dieting, and eating disorders. Evidence for ‘Westernization’s’ purported role in engendering Eds in Asian populations includes research from Singapore [38, 52], Hong Kong [53], Fiji [13, 54], Pakistan [26, 27], and Taiwan [55, 56], indicating a correlation between degree of Westernization and elevated body image disturbance.

### A closer look at patterns and trends by country

#### Singapore - trim and fit and mixed support for acculturation hypothesis

From a timeline perspective, EDs became increasingly common in Singapore throughout the 1990s surfacing alongside their emergence in neighboring Malaysia and Hong Kong [31]. Several studies also suggest that body dissatisfaction had grown as pervasive among certain segments of the population, such as university students and Singaporean Chinese schoolgirls, as it is in the West by as early as 2003 [38, 57, 58]. That said, the prevalence of documented clinical EDs was still thought to be relatively lower than in the West. Rates of EDs are forecast to continue to climb based on data from surveys of clinical samples, though this prediction would stand to benefit from current epidemiological data, which unfortunately is unavailable [59].

Lee et al. [57] explored the manifestation of different patterns of EDs according to ethnicity in an eight-year retrospective review of individuals with AN seen at an ED clinic between 1994 and 2002. Although a large majority of the cases were female (91.3 %), males accounted for 8.7 % of patients seen during that period. Results showed that Malays (4.8 % of cases) were underrepresented among AN cases in Singapore, as compared to individuals of Chinese (84.1 %) and Indian (7.9 %) descent [57]. In contrast, ethnic Malays appear to account for a significantly larger proportion of bulimic and binge eating disorder cases [60].

A particular social experiment carried out in Singapore during the 1990s is noteworthy. Aimed at addressing the rising rates of childhood obesity, the government established a compulsory school-based weight-loss program called “Trim and Fit” (TAF). The scheme was a failure in terms of addressing childhood obesity, and may have even contributed to ED onset in vulnerable individuals, based on a later study’s finding that 11.1 % of the AN patients seen during the 1994–2002 period were former members of the program, and participants in the TAF club reported experiencing social stigma and teasing from their peers [57], both of which are risk factors for EDs. Meanwhile, two recent cross-cultural studies comparing Singaporean women to Australian women, both with and without EDs, yielded inconsistent results. While one study revealed a significant association between greater acculturation to Western culture and increased body image disturbance [52], the second study found no significant relationship between acculturation and eating pathology [43, 52]. Authors of the latter of these two studies attribute the discrepancy in these findings to the first study’s reliance on attitudinal sub-scales of the EDE-Q rather than the full measure, which may have obscured important differences between cultural groups.

#### Hong Kong, India & non-fat-phobic AN

The single-most significant contribution of studies of EDs in Hong Kong from the early- to-mid- 1990s was the identification and documentation of a variant form of AN in which the hallmark features of AN in the West, namely ‘fat-phobia’ and/or distorted body image, were conspicuously absent [4, 61]. In such cases, patients typically attributed their restrictive food intake to somatic complaints such as epigastric bloating, abdominal/stomach pain, or an absence of hunger/appetite [61]. Compared to “typical” AN patients, individuals with non-fat-phobic Anorexia Nervosa (NFP-AN) manifested bulimic symptoms less frequently and tended to have lower pre-morbid BMI [35, 51–53]. In the years since then, considerable research has attempted to elucidate this ‘alternative’ form of AN, which contested the previously held assumption that a ‘fear of fatness and/or weight gain’ was the essential, defining characteristic of individuals with AN. What is dramatic; however, is that over time reports of NFP-AN cases in HK have steadily declined while BN and fat-phobic AN have steadily increased. Thus, individuals presenting with AN in Hong Kong today bear far greater resemblance to AN patients seen in the West, as opposed to their non-fat-phobic predecessors [35], with ‘desire for thinness’ and levels of body dissatisfaction continuing to grow more ubiquitous [54–56].

Beyond Hong Kong’s city limits, non-fat-phobic anorexia (AN) has also been reported in other Asian countries, such as India, although its occurrence is less frequent than typical AN and cases of NFP-AN have grown increasingly rare over time. In the context of India, eating disorders paint a particularly complex picture both historically and at present, perhaps reflecting the enormous diversity of its populace. Early reports of EDs began appearing in the mid-1990s, including five cases of young, single, Hindu women (aged 15-22), who exhibited persistent vomiting, amenorrhea, refusal to eat, significant weight loss, and numerous somatic complaints without particular import placed on thinness or fat phobia [66, 67]. Likewise, a study from 1995 involving 210 Indian university students, split nearly equally between males (*N* = 104) and females (*N* = 106), determined that 14.8 % of the sample evidenced Eating Distress Syndrome (EDS), a unique variant and precursor to clinical EDs [67]. In both of these studies, some of the patients did not evidence any detectable ‘fat phobia’ or ‘fear of weight gain,’ much like their counterparts in Hong Kong around this period of time.

#### Fiji, Pakistan & Taiwan and the role of media

Although technically located in Oceana, the cluster of islands that comprise ‘Fiji’ have contributed considerably to our understanding of the emergence and rise of EDs in non-Western countries. Consistent with many pre-industrialized countries, traditional Fijian notions of beauty upheld a heavier, more robust female body ideal, and as a result, EDs were quite rare, with only one case of an ED documented prior to the mid-1990s. What’s more, the islands were largely isolated from Western influences – specifically, Western media – until TV’s introduction into Fijian society in the late 1990s. In this way, Fiji has provided a real-world laboratory in which the thesis of ‘Westernization’ has been tested. Anne Becker and colleagues have conduced innovative studies measuring eating psychopathology and beauty ideals, both before and after the introduction of TV and found that in the wake of TV’s arrival on the island, rates of eating disturbances surged among ethnic Fijian women over the course of the next decade. Definitions of female beauty and body ideals within the broader Fijian society were also reconfigured in the image of a more ‘Westernized’ ‘thin ideal’ [13, 54, 68].

The studies documenting the emergence and proliferation of eating disturbances in Fiji began in the late 1990s and continued throughout the first decade of the new millennium [6, 69]. Around the same time, Fiji was beginning to industrialize. ED risk factors including increases in body dissatisfaction, dieting, desire to lose weight, thin ideal internalization, and disordered eating behaviors including binge-eating and self-induced vomiting, continued to become more widespread [54, 69, 70]. Acculturation has been posited as a key mediating factor in light of one study’s findings, which revealed a significant association between binge-eating and acculturated body attitudes [71]. Becker [13] suggests that Fijian females’ explicit modeling of their behavior and appearance on Western TV characters may reflect a desire to position themselves competitively within a rapidly changing culture. The most current research from Fiji shows that EDs continue to increase, as do reports from males who describe experiencing sociocultural pressure to achieve a muscular body ideal [70]. Additionally, it also appears contemporary social media may exert further negative influence on eating pathology among Fijians [72].

The media has likewise been implicated in influencing risk of ED development in studies conducted in Pakistan [73, 74] and Taiwan [41]. In many ways, Pakistan is a country that straddles the indeterminate cultural demarcation drawn between ‘East’ (as in Asia) and ‘Middle East’, which shares a long-standing but complex relationship with the Western world. Early reports of EDs appear in the 1990s in Pakistan [75], despite extant malnutrition and a historical association of female robustness with wealth and authority [73]. Studies focusing on schoolgirls were conducted in Mirpur, identifying one case of BN using DSM-III-TR criteria [27], and in Lahore, where a combined prevalence of 1.6 % for full- and partial EDs was found, as was some indication that more ‘Westernized’ participants faced greater risk of ED onset [26]. More recent data culled from studies involving university-level students report elevated risk for EDs [74, 76], and estimate that a third of university women (33.73 %) reported experiencing dissatisfaction with their weight [77]. Furthermore, a study of females aged 16 to 20 published earlier this year, found that 64.9 % of respondents scored 2 or above on the SCOFF questionnaire [78], placing them at risk for developing an ED.

From a media standpoint, Pakistan has historically been a more conservative, closed society with less exposure to Western media, fashion and advertising, as compared to many other countries in Asia. This has begun to change in recent decades however, concurrent with Pakistan’s increasing industrialization and urbanization. Accordingly, results from two 2011 studies suggest that media exposure correlates with negative body image and body dissatisfaction among both males and females [73, 79]. In fact, there is some reason to believe that males in Pakistan and elsewhere in Asia may face growing risk of developing EDs. A 2008 study of Body Dysmorphic Disorder in a sample of 156 Pakistani medical students (57.1 % female) revealed that a full 78.8 % of students evidenced some degree of body dissatisfaction, with ‘being fat’ ranking first among female students’ areas of concern (40.4 %) and second among males (32.8 %). Further analysis showed that while females were significantly more concerned about being fat (*p* = 0.005), males were significantly more concerned about being thin (*p* = 0.01) [80].

While concern about weight and shape may be on the rise among men in some areas of Asia, results from a study comparing body image among 55 heterosexual Taiwanese males to results obtained from identical studies conducted in the U.S. and Europe, suggests body dissatisfaction is less common among males in Taiwan [81]. Seeking to understand this outcome, the authors then analyzed the number of undressed male and female models in American and Taiwanese women’s magazine advertisements, respectively. They found that while American magazine ads frequently depicted undressed Western men, undressed Asian men were rarely portrayed in Taiwanese magazines, leading them to hypothesize that the pressure associated with male body ideals may be less pervasive at this point in time in Taiwan.

On the whole, data from Taiwan suggest that clinical EDs are less common than in Western countries. However, there is an abundance of research indicating that risk factors associated with ED development, such as body dissatisfaction and dieting, are increasing and are now commonplace among adolescents and young adults [39, 41, 82]. As was found in Pakistan, two studies conducted in Taiwan indicate that the landscape of disturbed eating pathology among a younger demographic is cause for concern. The first study reported elevated EAT-26 scores among 17.11 % of the 1605 high school students surveyed [45], while the second – a cross-sectional survey of 835 female junior high school students – found 10.4 % of students to be at elevated risk for an ED based on EAT-26 scores exceeding 20 [83].

Delving deeper than identifying at-risk individuals, Liou and colleagues [84] investigated methods of weight control used by 15,716 adolescents (male: 7043; female: 8673) ages 10-18 years spread across 120 representative schools. These schools were selected using a three-stage stratified systematic sampling design with probability proportionate to the size of the population in Taiwan, in an effort to collect nationally representative data. Results showed that younger adolescents were more likely to engage in purging behaviors, and that such behaviors were linked to factors including frequent daily media use (e.g. TV, internet, etc.), consumption of fried foods, and night-time snacking [84]. In regards to media exposure – which serves as a conduit for thin-ideal messages – research with Taiwanese adolescents showed that it fosters greater internalization of the thin ideal and experiences of ‘media pressure’ to be thin, both of which were found to significantly increase body dissatisfaction; in turn, ‘media pressure’ and body dissatisfaction were identified as the factors which significantly contributed to restrictive eating behaviors and unhealthy weight-control methods, even after all other variables were controlled for [56].

#### Japan & steady rise in eating disorders

Contemporary understanding of EDs in Japan has its roots in investigations that began in the 1970s and continued over the subsequent two decades, which collectively, show a consistent rise in ED prevalence and incidence during this period. Despite this trend, however, EDs remained less common in Japan than in industrialized Western countries. Most of the evidence from this period is derived from clinic-based studies, some of which focused on anorexia (AN) exclusively, as community-based data was minimally available. On the whole, these studies demonstrate a continual increase in reported cases of EDs in Japan during the latter half of the twentieth century [85]. Much like in the West, ED cases were highly concentrated among adolescent females, with prevalence estimates ranging from 25.2–30.7 (per 100,000) among females 13–29, as compared to 6.3–9.7 for the entire female population, and 3.6- 4.5 among the general population [86]. Cases of eating disorders among males, although rare, have been documented in Japan [87]. In their 2010 review of the literature from Japan, Chisuwa & O’Dea note that rates of EDs among males in non-clinical settings were thought to be around 2–3 %, whereas rates reported for Japanese female adolescents ranged from 5–10 % [88]. More research is available regarding body image concerns among Japanese males, with studies reporting that male adolescents often desire to gain weight, frequently underestimating their current body weight, and experience rising body satisfaction as they acquire more muscularity with age [132].

With regards to bulimia nervosa (BN) and binge-eating, a study of college women carried out in the 1980s indicated that 8.4 % of participants engaged in binge-eating and self-induced vomiting, including a subset of whom (2.9 %) reported purging more than once weekly. Meanwhile, 4.6 % of women reported using purgatives as a weight-control strategy [89]. Rates of EDs surged dramatically in the first decade of the millennium, with cases of AN increasing four-fold and BN prevalence rising to 4.7 times its 1990s-era levels [90]. As Pike and Borovoy (2004) [24] note, the proliferation of EDs in Japan coincided with increasing industrialization, urbanization, and globalization, and this confluence of factors served to catalyze significant changes in gender roles as well as the traditional Japanese family structure – a pattern that would be replicated in developing countries across the globe. Today, more recent epidemiological data from Japan indicates that EDs are now on par with rates in many industrialized, Western countries, but consistent with trends observed of late in Western countries, ED rates appear to be relatively stable rather than increasing at present [87].

Intriguingly, a recent study of women in Japan – where conventional AN has historically been the norm – suggests that unique subtypes and differences among individuals with AN may be even more multi-layered than was previously thought. Through structured clinical interviews and administration of the Eating Attitudes Test (EAT) and Eating Disorders Inventory (EDI), the authors identified three discernible groups of anorexic patients: 200 women (52.2 %) were diagnosed with typical AN; 86 (22.5 %) with non-fat-phobic AN (NFP-AN); and 97 women (25.3 %) with AN who displayed no evidence of distortions related to body shape and weight (AN-NED). The groups differed significantly on factors including duration of illness, maximum and minimum BMIs, and AN subtypes, as well as EAT scores, EDI totals, and all subscales of the EDI [90]. Thus it seems that the clinical presentation of EDs, and specifically AN, is dynamic and continuously evolving, much like Asia’s rapidly transitioning economies.

#### South Korea, China and Thailand: eating disorders symptoms can outpace western comparison groups and challenge model of westernization

Like other countries in Asia, increases in rates of clinical EDs and associated risk factors were observed in South Korea in conjunction with a period of pervasive societal change from the early 1960s through the late 1990s, which was further buoyed by a rapidly accelerating economy. According to a 1998 epidemiological study (*N* = 3062) of Korean adult Men (*N* = 1249) and women (*N* = 1813), 8.5 % of those surveyed evidenced abnormal eating pathology, as determined by scores above the cut-off (>20) on the Korean version of the EAT-26 (K-EAT-26) [40]. Overall, reports of EDs from Korea portray a clinical profile very similar in presentation to cases observed in the West. Moreover, recent findings suggest that body dissatisfaction and internalization of the thin ideal may, in fact, be more widespread in Korea than in the West. For instance, a study that compared eating pathology as measured by the EAT-26 between 167 s-generation female, young adult Korean-Americans, with 37 Korean immigrant women (immigrated in last 7 years), and 937 native Korean women, found that Korean-American women scored significantly lower than both Korean immigrants and native Koreans. Meanwhile, Korean immigrant and native Korean women’s scores did not differ significantly from one another, even though native Korean women reported significantly lower BMIs than their Korean immigrant counterparts. Likewise, degree of ‘acculturation’ in both immigrant and native Korean women, which was measured using the Suinn-Lew Asian Self-Identity Acculturation Scale (SL-ASIA), did not bear a significant relationship to their EAT-26 scores [91]. Rather than solely crediting Western beauty ideals for perpetuating an unhealthy ‘thin’ archetype, Jackson, Keel & Lee suggest that native Korean values may also promote eating disorders, owing to their emphasis on appearance rather than ability or talent as the factor crucial to a woman’s success in marriage and in career [91].

In a study comparing college women from China (*n* = 109), Korea (*n* = 137), and the U.S. (*n* = 102), Korean college women evidenced the greatest degree of body dissatisfaction and disordered eating behaviors, followed by Chinese women, and lastly, U.S. women. Intriguingly, a similar outcome was observed in a cross-cultural study comparing eating-related attitudes and psychopathology among Thai (*n* = 101), Caucasian Australian (*n* = 110) and Asian Australian (*n* = 130) students at a university in Australia. Thai students scored significantly higher on both the EAT-26 and Eating Disorders Inventory -2 (EDI-2) than their Australian Asian and Caucasian Australian counterparts, who did not differ significantly from one another [92]. In both studies, these findings are inconsistent with the premise of the ‘Westernization’ model of EDs, which would predict that U.S. women would exhibit the most eating-related dysfunction [15].,

#### Malaysia and cultural diversity within national borders

One of the earliest prevalence estimates for EDs in an Asian country is from a 1981 study, conducted in Malaysia, which extrapolated a national prevalence estimate of 0.05 % for AN, based on a sample of 6000 psychiatric patients [30]. A second wave of studies from this region arrived nearly twenty years later, at a time when Malaysia was experiencing a flurry of economic development and concurrent increases in both population BMI and rates of body dissatisfaction and dieting behaviors [93]. Viewed collectively, the research from Malaysia provides a solid base of evidence for implicating gender and ethnicity as primary factors influencing ED risk.

Regarding significant gender differences, the research suggests that in comparison with males, females often exhibit greater body dissatisfaction and concern surrounding eating and weight/shape issues; show a preference for very slim or even underweight ideal body figures; and employ a range of weight-loss strategies, including dieting, self-induced vomiting, exercise, and laxative abuse [8, 94, 95]. Males, on the other hand, are more likely to perceive themselves as overweight but fail to perceive if they are underweight, consider an overweight figure to represent the male body ‘ideal’ [94], and report engaging in activities intended to build muscle [8]. In another study of 584 male and female university students, roughly one in five students (18.2 %) were at heightened risk for EDs, with females facing greater risk than males (21.3 % vs. 13.5 %) and reporting higher stress scores [96].

Malaysia represents one of the most ethnically diverse countries in Asia, and thus, several studies have compared EDs among ethnic Malays (Malaysian Malays), and Malaysian Chinese and/or Malaysian Indians. For instance, results from an analysis of eating attitudes among 187 ethnic Malays (*M* = 87) and 80 Chinese students (*M* = 33) attending a Malaysian university, indicated that Malay students were at greater risk for ED onset as compared to fellow Chinese students, based on scoring significantly higher on the EAT-26 [97]. Another study, also comparing Malaysian (*n* = 459), Chinese (*n* = 307), and Indian (*n* = 150) women, assessed whether ethnicity influenced the discrepancy between actual vs. ideal weight. Indeed, Chinese women evidenced less discrepancy between ideal vs. actual weight than did Malay and Indian women, however the effect size was small [95]. Study findings also lent some support to the premise that women may internalize appearance-based media messages differently according to their ethnicity.

Conversely, a 2013 study comparing adolescents of Malaysian Malay (*n* = 58), Malaysian Chinese (*n* = 95), Chinese native (*n* = 242), and non-Asian Australian backgrounds (*n* = 81), reported that Malaysian Chinese adolescents had the greatest degree of body dissatisfaction. In particular, it was found that overall body dissatisfaction was positively correlated with dissatisfaction with facial features among Malaysian Malays and Australians [11] – a finding that is consistent with traditional Asian beauty ideals that focus on facial features. The diversity within Malaysia is an important reminder that as much as there are cultural trends, in many countries there will be significant variations within country based on factors such as ethnicity.

#### China’s late arrival but significant rise in eating disorders

The story of EDs in China represents a more recent and less developed chapter in the rise of EDs in Asia, driven in part, by the fact that Mainland China embarked on the path of industrializing and modernizing slightly later than some other Asian societies [98]. As was noted earlier, initial reports of EDs in China surfaced in the early 1990s, including cases of BN in two females and one male [99], and a case of early-onset AN in a 4-year-old Chinese boy [100]. In each case, the individual exhibited the hallmark ‘fear of fat’, which served as the underlying driver of their ED. Some of the evidence from China indicates that Asian males may face a heightened vulnerability to developing EDs as compared to males in the West. Some have hypothesized that the presence of ‘fear of fat’ in cases in China may stem from the fact that it is one of the few non-Western societies in which there exists a historical precedent for valuing thinness in women, as it was deemed a desirable trait of femininity [42, 101].

Other explanations offered for these findings have included many of the same factors implicated in the emergence of EDs in other Asian countries, namely Westernization, mass migration from rural to urban city-centers, and after-effects of sociocultural transformation like economic stress, and competition, and the disruption of social and emotional support. Research from China identifies several factors that increase overall vulnerability to EDs, including higher SES [102], concerns about body shape and size and preference for a thin body-ideal (esp. in girls) [103], a history of child abuse, elevated anxiety levels, acrimonious relations with parental figures, concerns related to media idols, and above-average scores on the body dissatisfaction and interoceptive awareness factors of the EDI [104]. Furthermore, Jackson and Chen [105] make a convincing case for the influence of environmental factors such as perceived pressure from mass media, interpersonal relationships, and fear of negative appearance evaluation, in the etiology of BN in adolescents. Other researchers have likewise identified the media as a perceived source of pressure relating to physical appearance, even positing that it may predict body change behaviors in both males and females [106, 107].

Our understanding of EDs in China has been advanced considerably by the publication of several larger community-based studies in the early years of 2000, which explored factors known to play a role in ED onset such as body image and eating behaviors, in male and female adolescents [106, 108, 109]. As a whole, these studies suggested that the incidence rates for clinical EDs were relatively low, however, they also provided indication that cases of subclinical and/or partial EDs were not uncommon, and risk factors such as body dissatisfaction and maladaptive eating behaviors, were in fact widespread. Indeed, some of the most recent studies indicate that BN is becoming more prevalent among young Chinese females [110, 111]. In illustration of this, prevalence rates of 1.05, 2.98, and 3.58 % for AN, BN, and BED, respectively, were estimated from a 2013 study of female university students in Wuhan, China. These findings are a cause for great concern as these rates rival those typically reported among similar groups in the West, suggesting that EDs are quickly becoming a formidable problem and public health challenge in China [112].

A 1999 study that assessed measures of disordered eating attitudes and behaviors, drew an interesting comparison between high school girls in urban HK and similar samples of girls residing in two locations within Mainland China: the largely rural Hunan province, and Shenzhen, a semi-urban, rapidly growing industrial center. The level of industrialization was found to mediate disordered eating attitudes and behaviors, such that girls in HK displayed the most pronounced eating disturbances and body dissatisfaction, followed respectively, by girls in Shenzhen and Hunan province [98].

#### India’s complex picture – eating disturbances in the context of low weight and shape concerns

Although findings from individual case studies [113], as well as larger studies involving male and female university students [114, 115], describe the emergence of familiar manifestations of EDs in India, the data also suggest that psychogenic vomiting is the most common ED diagnosis in India (approx. 84 %), most often observed in individuals from middle or lower-SES families, who are first-born or only children, and in whom illness onset precedes puberty. By comparison, a diagnosis of AN (14.6 % of all ED diagnoses), was associated with an upper-SES background, not being the first-born, and ED onset in adolescence. Therefore, although there may be reason to believe that factors contributing to ED onset such as body dissatisfaction and dieting, may be on the rise, it is important to note that available evidence does not suggest that unhealthy weight loss strategies and eating disordered behaviors are common in India [114–117], nor does it give any indication that rates of clinical EDs are rising [118]. Even so, the perception that EDs are increasing in urban India appears to be gaining ground, based on the findings of a 2012 survey of 66 practicing psychiatrists whom were asked whether they perceived EDs to be a ‘serious clinical issue’ in India. Collectively, a total of 74 ED cases – including two males – were reported based on accounts from 45 participants who indicated having seen patients with EDs in the past 12 months, which included 32 instances of AN, 12 of BN, and 30 of EDNOS, respectively. Interestingly, 23.5 % of respondents believed that rates of EDs were rising in Bangalore, while 26.5 % surmised that rates were holding steady, the largest group – 42 % -- expressed uncertainty [119]. In general, some of the data from India contradicts the hypothesis that acculturation moderates eating pathology (e.g., Bhugra, Bhui, Gupta, 2000) [125], while others report results that suggest urbanization and SES (which is often correlated with acculturation) are associated with elevated risk for body weight dissatisfaction and dieting [120, 121]. In light of the sheer physical size of India and the considerable diversity of its populace, it seems probable that trends may vary among distinct segments of the Indian population and much further research is needed.

#### Thailand – clinical case report from rural, low-SES region

While fewer studies of EDs in Thailand are available as compared to many other Asian countries, a case of restrictive AN reported in a 13-year-old Thai female is intriguing on account of the fact that the girl was from Thailand’s poorest province (Khon Kaen) [122]. Differences in beauty ideals and body shape/weight concern among Thai women typically reflect the influence of rural vs. urban settings, such that women from more developed, urban regions, place greater emphasis on body weight and shape in assessing physical appearance identity [123]. Further evidence of vulnerability to EDs comes to light in a study of 447 female undergraduates in which 6.34 % of students reportedly obtained high EAT-26 scores (>20). Consistent with findings from other regions of Asia, 19.25 % of respondents exhibited body image dissatisfaction (BSQ scores > 110) despite a majority (56.17 %) of the sample having ‘normal’ BMIs [124].

#### Deconstructing “Westernization” and incorporating urbanization and industrialization

In deconstructing the usage of the term “Westernization” within the context of Asian economic development, it is important to acknowledge that many of the characteristics that have been ascribed to “Westernization” may, in fact, be more accurately associated with the processes of industrialization and urbanization, irrespective of countries of origin. While the “west” experienced industrialization and urbanization earlier in history, that process now unfolding throughout Asia may best be thought of as a new wave of industrialization and urbanization, moving beyond the idea that the wholesale export of Western culture and influence, broadly defined, is the variable to which we should continue to devote primary attention when examining cultural factors associated with the rise of EDs in Asia. Consistent with Omran’s hypothesis, industrialization and urbanization are correlated with other social and cultural shifts such as improved access to biomedical care, eradication of malnutrition and poverty and increased affluence. Although positive overall, these developments trigger new challenges, namely non-communicable disease such as EDs. Given the range of pathways in the emergence of eating disorders in Asia, we will learn more about the relationship of culture to eating disorders if we recognize that eating disorders are not culture-specific or culture bound, but perhaps better thought of as culture-reactive, whereby certain cultural contexts result in social and environmental changes that increase risk for EDs.

What is beyond question is the fact that the world population is becoming more urban, and in Asia, this process of urbanization, driven by industrialization, has been especially dramatic due to the sheer size of the population and the rapid speed with which it has occurred. While not unlike the first wave of Western industrialization and urbanization, some of the consequences of these profound societal transformations are positive, such as increases in per capital income [16], however, Asian industrialization and resultant urbanization have also been linked to increases in the overall rate of psychopathology [125], including EDs, throughout the region. This is consistent with data from the Netherlands that indicated that the incidence of BN was correlated with degree of urbanization, with BN rates five times higher in urban centers as compared to rural areas. Anorexia nervosa (AN), however, was not associated with urbanization [14]. Meanwhile, in China, data suggest that intensifying urbanization corresponds with increases in overall eating pathology, as characterized by decreasing BMIs, increasing weight and shape concerns and increasing dieting behaviors [98].

Again, independent from the so-called “Westernization” effect*,* data from countries across the globe substantiate that the move from rural to urban living has been shown to coincide with drastic changes in diet and a shift towards a more ‘sedentary’ lifestyle. During the ensuing ‘nutrition transition’ from farms to fast food, more prepared and processed foods – foods that have high fat, sugar, and salt content - have steadily crept into Asian diets that were traditionally rich in vegetables, fiber, and grains [17]. In addition to urbanization broadly defined, other correlated factors found to precipitate and facilitate this dietary shift include rising income as well as the transition to a wage economy, changing gender roles, and an overall increase in the accessibility of food [126]. The proliferation of American fast food chains, which first arrived in Japan in the 1970s, and eventually spread throughout all of Asia are quintessential examples [127]. Unfortunately, increased consumption of these foods serves up a supersized side order of “Nutrition-Related Non-Communicable Diseases” (NRNCD) including obesity, hypertension, diabetes, and other metabolic disorders [17]. Substantiating this trend, population weights and obesity prevalence across Asia are steadily climbing and have become significant public health concern in Asian societies, including but not limited to Japan [128], South Korea, Malaysia and China [18, 129–130].

#### Sociocultural transition and gender roles as related to industrialization, urbanization and eating disturbances

Among the manifold changes set in motion by the processes of industrialization and urbanization, there also exists ample evidence of a profound and dramatic shift in gender roles that occurs within societies undergoing transition. This shift marks one of the most significant contextual factors in the rise of EDs across diverse Asian societies, uniting concurrent forces of economic pressures, a developing consumer culture including globalized fashion and beauty industries, as well as media influence and acculturation. This pattern, which has been observed consistently across Asia, has a particularly transformative effect on the landscape of women’s lives. Specifically, Witcomb, Arcelus and Chen [101] suggest that the demands of an increasingly competitive environment in which women are expected to develop a new set of skills, may unavoidably expose them to greater criticism from peers, colleagues, and society, perhaps in turn prompting women to engage in more self-evaluation. Thus, physical appearance becomes one of several domains in which women ‘measure’ themselves against an aspirational ideal [101]. In Asia, the ‘gendered’ nature of societal transformation and globalization is especially striking due to the fact that economic development was initially driven by the growth in the manufacturing (specifically garment) industry, and later, the service industries, which resulted in a spike in demand for women’s labor in particular [19].

Unsurprisingly, with growing numbers of women pursuing employment and educational opportunities, familiar definitions of “femininity” and conceptions about gender roles are called into question and traditional family structures are subject to change as well. During such times of rapidly changing gender roles, Striegel-Moore, Silberstein and Rodin (1986) [131] suggest that girls, in particular, are very likely experiencing the stresses of these shifting roles and evolving expectations, which in turn, may place them at greater risk for overall psychological distress [131]. Similarly, changes in male gender roles may be contributing significantly to the increases in rates of EDs among males.

### Review & future directions

This discussion attempts to outline and contextualize the emergence of EDs in Asia by locating this development within the broader discourse surrounding the processes of change that have radically transformed the region in the last three decades. With the ‘Westernization’ thesis serving as the foundation, adding the constructs of industrialization and urbanization allows for a more complete and thoughtful analysis of EDs in Asia. The construct of “Westernization” bestows assumptions of primacy, power, and agency on ‘Western’ culture by conceptualizing it as ‘exportable’, while the “recipient” cultures are constructed as immobile and fixed [37]. Indeed, overly exaggerated notions of ‘Westernization’ conflate, or fail to account fully for, concurrent processes of societal transformation such as the current wave of industrialization and urbanization now occurring in Asia. The danger of subsuming these respective phenomena under the umbrella of ‘Westernization’ is that it over-attributes the diverse changes and distinct developmental pathways of individual Asian societies, including the emergence of EDs, to the influence of the West. As variations in the expressions of EDs across cultures in Asia make clear, individual Asian societies have not responded uniformly to complex global shifts and pressures, nor have they passively absorbed Western templates of beauty.

One challenge researchers face is that many of the measures and criteria typically used to assess eating disorder psychopathology are based on Western models and have not been fully validated for use with non-Western samples. Progress is being made and the translation and evaluation of these assessment instruments for use with diverse groups is increasing (e.g. [27, 40, 132, 133]). By analyzing the multifold ways in which a more inclusive range of significant societal phenomena may impact the emergence and/or spread of EDs, we have sought to shed light on how the changes within societies affected by industrialization, urbanization, and ultimately, globalization, have a unique impact on adolescent girls and young women in particular. Consequently, women in cultures undergoing transition inhabit a tenuous position as they seek to navigate a landscape characterized by changing definitions of femininity, gender roles, women’s increasing integration into the public sphere and involvement in emerging labor forces, and rising sociocultural pressure to conform to new aesthetic beauty ideals that glorify thinness.

Building upon previous research that has linked the emergence and rise of EDs with cultures undergoing transition, we propose that the cumulative effects of societal changes including the proliferation of media images promoting thinness and a consumer culture dominated by globalized fashion and beauty industries, are placing individuals, especially women, across Asia at risk for developing EDs. Faced with uncertainty, paradoxical messages, and stress, those with individual risk factors or underlying vulnerabilities to EDs may ultimately join the growing ranks of women and men suffering from these disabling disorders. Increases in ED cases in respective Asian countries will likely correspond to the stage of societal transformation in a particular society is in with nations further along the developmental continuum exhibiting the highest rates of EDs.

Our examination of the pathways by which EDs have emerged and socioeconomic transitions that have transpired within Asia, has in turn necessitated a deconstruction of this geopolitical label. From both a historical and contemporary perspective, the individual societies that comprise ‘Asia’ exhibit marked diversity both in relationship to one another as well as in relation to the larger global community. Recognition of the distinctiveness of these individual cultural and national identities is crucial to understanding the emergence and rise of EDs across these varied contexts.

## Conclusion

Within the broader field of eating disorders, it is important to recognize that the study of EDs in Asia, specifically, is still in its early stages and that research is limited, albeit quickly expanding [134]. Future epidemiological research and community and clinical studies are needed to further advance our understanding, prevention, and treatment of these disorders within the context of the various cultures in Asia. Bearing in mind then the considerable heterogeneity and diversity of cultures subsumed under the ‘Asia’ umbrella, it is particularly important that future studies examine eating disorder psychopathology not just within each of the individual countries in Asia, but also in relationship to one another, as well as in broader cross-cultural studies. Studies that are more broadly representative of a particular society, and which include males, minority ethnic groups, and a wider age range of participants, are needed if we are to discern a truly complete picture of eating disorders across cultures. Cross-sectional studies suggest that risk factors for eating disorders among males are largely similar to those identified among females, such as body mass index, negative affect, self-esteem, perfectionism, drug use, perceived pressure to lose weight from parents and peers, participation in sports that focus on leanness, and disturbances around the pursuit of muscularity among adolescent boys [135]. These data are largely cross-sectional, and further studies exploring risk for eating disorders among Asian males is extremely important given the sheer numbers of individuals this represents.

Fully validated and culturally appropriate assessment instruments and design methodologies are needed, as is a more flexible, adaptable approach to the application of diagnostic criteria derived from Western samples within non-Western settings. Furthermore, a critical aspect of the emergence and spread of EDs in Asia is that it has transpired concurrently with rapidly rising population BMIs and rates of overweight and obesity across the continent. Indeed, similar to many Western countries, obesity rates have reached epidemic proportions in Malaysia, Singapore, Thailand, and other parts of South Asia [18, 129, 130]. Although EDs and obesity have historically been treated as independent public health issues, they share many common etiological factors, and successful strategies for combating one will almost certainly impact the other. Thus today, we are presented with a critical and time-sensitive opportunity to address two serious public health crises. Success in this endeavor will be contingent on researchers and policymakers in both arenas working in partnership with one another to develop effective and innovative strategies around prevention and intervention of EDs and obesity.

As the conversation within the field of eating disorders shifts away from affirming ‘Westernization’ as the cornerstone of explanations for the rise of EDs both in Asia, and across the developing world, what we see in many ways is the receding power of (or ‘emphasis on’) the West and the concurrent, unabated transcendence of globalization. Fueling this transition is growing recognition that the independent processes of societal change – industrialization and urbanization – are not a byproduct or unique export of Western culture, but rather are natural steps along a society’s pathway of economic development. Thus, as the countries on the Asian continent continue to advance along the spectrum of development, we cannot assume that their independent trajectories – and associated patterns of EDs and obesity rates – will track a course identical to that observed in the West. We can, however, expect that these continued processes of economic development will play a significant role in impacting rates of EDs across the region. Just as the transformation of Asian countries on political, social, and economic terms has given rise to unique societies that differ in significant ways from the industrialized West, so too, will the course of EDs in Asia chart a distinct course that will contribute meaningfully to our global understanding of eating disorders.
